# Self-Oligomerization Is Essential for Enhanced Immunological Activities of Soluble Recombinant Calreticulin

**DOI:** 10.1371/journal.pone.0064951

**Published:** 2013-06-10

**Authors:** Shang-Hui Huang, Li-Xiang Zhao, Chao Hong, Cui-Cui Duo, Bing-Nan Guo, Li-Juan Zhang, Zheng Gong, Si-Dong Xiong, Fang-Yuan Gong, Xiao-Ming Gao

**Affiliations:** Institute of Biology and Medical Sciences, Soochow University, Suzhou, Jiangsu Province, China; King’s College London, United Kingdom

## Abstract

We have recently reported that calreticulin (CRT), a luminal resident protein, can be found in the sera of patients with rheumatoid arthritis and also that recombinant CRT (rCRT) exhibits extraordinarily strong immunological activities. We herein further demonstrate that rCRT fragments 18–412 (rCRT/18-412), rCRT/39-272, rCRT/120-308 and rCRT/120-250 can self-oligomerize in solution and are 50–100 fold more potent than native CRT (nCRT, isolated from mouse livers) in activating macrophages in vitro. We narrowed down the active site of CRT to residues 150–230, the activity of which also depends on dimerization. By contrast, rCRT/18-197 is almost completely inactive. When rCRT/18-412 is fractionated into oligomers and monomers by gel filtration, the oligomers maintain most of their immunological activities in terms of activating macrophages in vitro and inducing specific antibodies in vivo, while the monomers were much less active by comparison. Additionally, rCRT/18-412 oligomers are much better than monomers in binding to, and uptake by, macrophages. Inhibition of macrophage endocytosis partially blocks the stimulatory effect of rCRT/18-412. We conclude that the immunologically active site of CRT maps between residues 198–230 and that soluble CRT could acquire potent immuno-pathological activities in microenvironments favoring its oligomerization.

## Introduction

Calreticulin (CRT) is a 46 kDa Ca^2+^-binding glycoprotein in the endoplasmic reticulum of eukaryotic cells [1]–[2]. It folds into 3 domains including a lectin-like globular N domain (amino acid residues 18–197), a proline-rich P domain (residues 198–308) and a Ca^2+^-binding C domain (residues 309–412) [3]–[5]. CRT can also appear at the surface of various types of cells exhibiting multiple biological functions [6]–[12]. Recently it has been shown that soluble CRT is present in the sera of patients with rheumatoid arthritis and with SLE [13]–[14] and that natural CRT (nCRT), isolated from human or mouse tissues, can directly activate macrophages in vitro [15]. Additionally, rCRT/39-272, a prokaryotically-expressed murine CRT fragment covering amino acid residues 39–272 fused with an N-terminal His-tag, was extraordinarily potent in activating B cells and macrophages in vitro and also in eliciting specific Ab production in mice [16]. This recombinant polypeptide also exhibited potent adjuvanticity, effectively assisting IgG production against conjugated target Ags with or without T cell help [16]–[17]. However, molecular mechanisms underlying this phenomenon are far from clear. Recent X-ray crystographical studies by Kozlov et al and Chouquet et al have defined the carbohydrate-binding site (involving residues Phe^74^, Met^131^, His^145^, Ile^147^ and the Cys^107^–Cys^137^) and also a peptide-binding site on its opposite side (Phe^74^, Trp^319^, Cys^105^–Cys^137^, Asp^135^) in the N domain of CRT [18], [19]. The sequence of rCRT/39-272 encompasses most of the globular N domain (aa residues 18–198), and we have previously shown that it possess lectin-like activity (selective binding with polysaccharides including carrageenan, alginic acids, and hyaluronic acids in ELISAs) [16], implying that the prokaryotically expressed recombinant polypeptide retained the lectin activity of CRT. It is of interest to determine if destroying the carbohydrate binding and/or peptide-binding sites (by deleting first half of the N domain sequence) would also abolish the potent immunological activities observed in rCRT/39-272.

Additionally, the fact that rCRT/39-272 is substantially more potent than nCRT in activating macrophages(see below) raised concerns regarding the possibility of LPS contamination in the *E. coli*.-expressed recombinant product. The “LPS contamination” hypothesis suggests tight binding between bacterial LPS and rCRT and also that, due to a synergistic effect, the LPS-CRT complex is a more potent immune activator than free LPS and rCRT alone. Based upon the observation that interaction between the N and C domains of CRT influences its structural stability as well as functional activity [20], a “C-domain deletion” hypothesis has also been postulated, suggesting that deletion of the C-domain (as in rCRT/39-272) leads to exposure of an immunologically active site (IAS) in the N and/or P domains of CRT. The present study compares the biochemical characteristics of nCRT and a series of truncated rCRT polypeptides and investigates the molecular mechanisms underlying the potent immunological activities of soluble rCRT. The results arising from this study have important implications for our understanding of the potential role of soluble CRT in immunopathological conditions.

## Results

### Biochemical Properties of rCRT and nCRT

**Figure 1:**
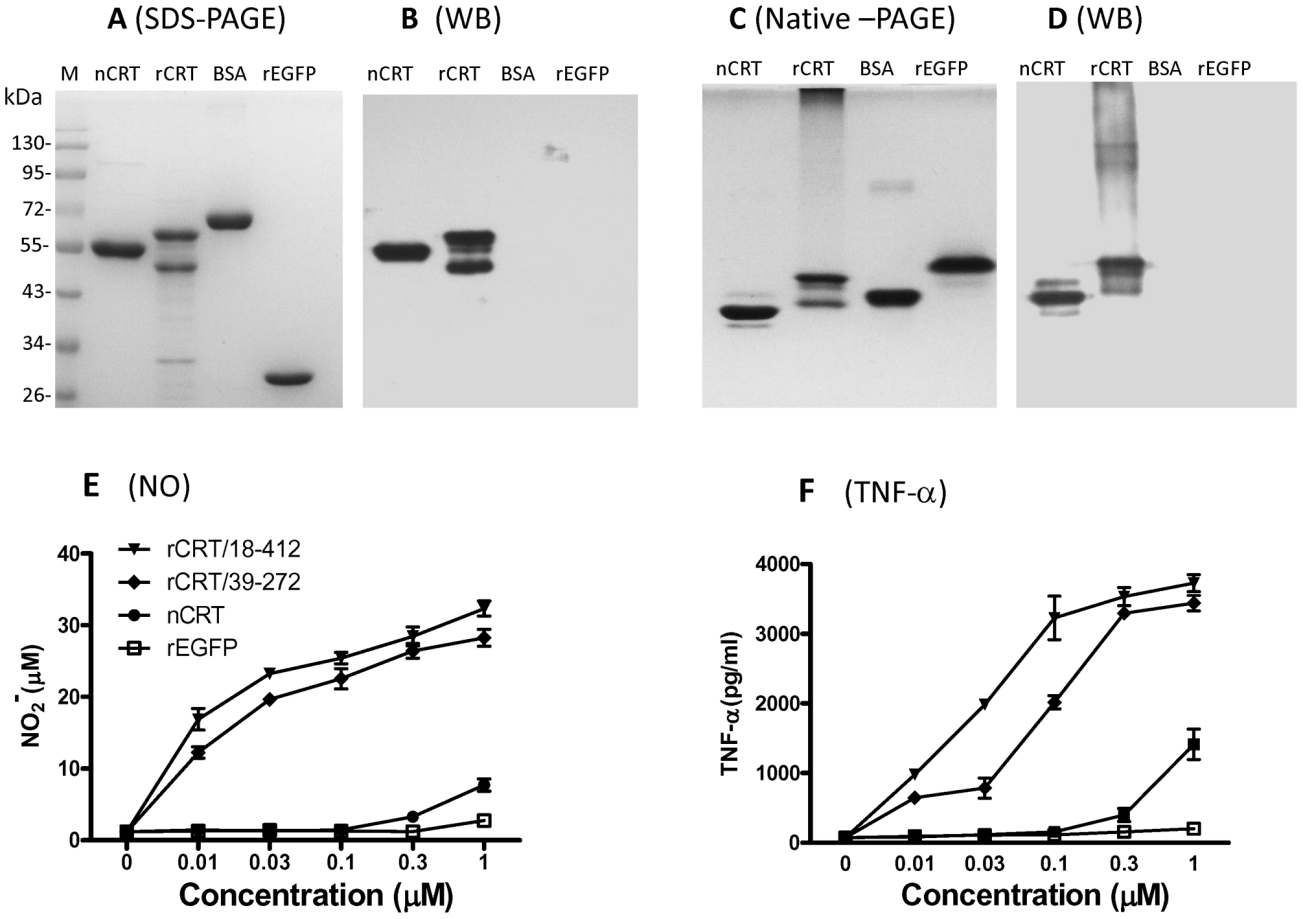
Preparation of nCRT and rCRT/18-412. (A) Pre-cleared mouse liver lysate (15 ml) was loaded onto a DEAE-A50 (10×2 cm) column and eluted using linear gradient NaCl 280 mM to 500 mM, collecting 1 ml fractions in cold room. Samples of input (lane 1), flow through (lane 2), 280 mM pre-wash (lane 3) and elution fractions (lanes 4–18) were analyzed by a SDS-PAGE 12% gel followed by CBB staining. Fractions 6–10 were combined, dialyzed against PBS, and then adjusted to 1 mg/ml for use as nCRT. (B) A Ni-column was employed for purification of rCRT/18-412 from a lysate of IPTG-induced *E. coli* harboring the expression vector for rCRT/18-412. Samples of *E. coli* before and after IPTG induction were loaded in lanes, flow through (binding buffer), wash through (20 mM imidazole), resultant rCRT/18-412 (300 mM imidazole), and stripping through were loaded, in the order of 1–6 into a SDS-PAGE 12% gel followed by CBB staining. Samples of nCRT, rCRT/18-412, BSA and rEGFP were compared in CBB-stained SDS-PAGE 12% gel (C) and native-PAGE (E), followed by WB using rabbit anti-CRT polyclonal antibody for detection (D, F). The secondary Ab was HRP-labeled goat-anti-rabbit IgG, with OPD as substrate.

Native CRT was extracted from mouse livers by (NH_4_)_2_SO_4_ precipitation followed by ion exchange chromatography on a DEAE-A50 column using a linear gradient of 280–500 mM NaCl for elution. Samples of the eluted fractions were assayed by SDS-PAGE (Fig. 1A), which showed that the eluent between 360–380 mM NaCl contained protein bands of the expected molecular weight for nCRT (55 kDa), which showed approximately 90% homogeneity judging by density of the major band in Coomassie blue (CBB)-stained gel (Fig. 1C).

Recombinant murine CRT fragment 18-412 (rCRT/18-412, with an N-terminal His-tag and without C-terminal KDEL) was expressed in *E. coli* and affinity purified using a Ni^2+^-column. The resultant rCRT/18-412 product contained 3 major protein bands at 60, 46 and 32 kDa (Figs. 1B & 1C); the two larger bands (namely rCRT-60 kDa and rCRT-46 kDa), but not the smaller one (Cp32), were recognized by polyclonal rabbit-anti-CRT antisera (CRT-Abs) in Western blot (WB) (Fig. 1D). Purified nCRT, but not BSA or recombinant enhanced green fluorescence protein (rEGFP, 28 kDa with a His-tag), was positively recognized by CRT-Abs. As evidenced by native PAGE analysis, a substantial amount of rCRT/18-412 formed higher-molecular-weight oligomers, whilst nCRT existed mostly in monomeric form (Figs. 1E & 1F).

### Lack of Specific Binding and Synergy between Soluble CRT and LPS

**Figure 2:**
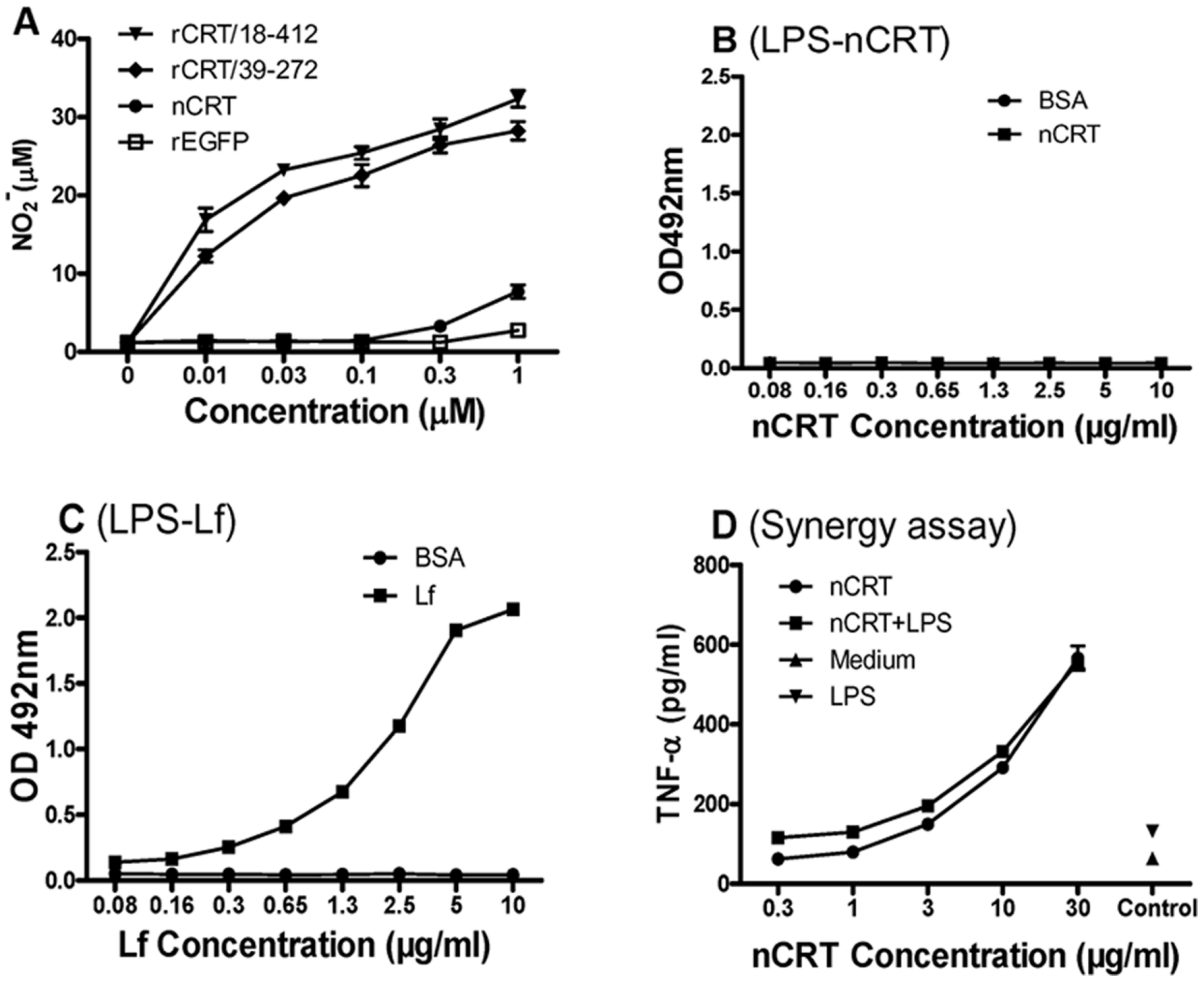
Lack of specific binding and sinergy between LPS and nCRT. (**A**) Freshly isolated murine peritoneal macrophages were stimulated with rCRT/18-412, rCRT/39-272, nCRT or rEGFP (0.01–1 µM) for 24 hrs. Concentration of NO_2_^−^ in the culture supernatant was then determined using Griess Reagent and the results are expressed as mean NO_2_^−^ concentration (µM) ± SD. LPS-based ELISAs were performed for detection of LPS binding with CRT (**B**) or lactoferrin (**C**). Lf or nCRT (2 µg/ml) were added to wells in polyvinyl plates pre-coated with LPS (10 µg/ml), with BSA as a negative control. Combination of polyclonal rabbit Abs against CRT, or lactoferrin, and HRP-labeled goat-anti-rabbit IgG was used for detection with OPD as substrate. The results are expressed as absorbance at OD492 nm±SD. For sinergy analysis (**D**), freshly isolated mouse peritoneal macrophages were stimulated with nCRT (0.3–30 µg/ml) in the presence, or absence, of LPS (0.1 ng/ml) for 24 h. Cells in medium alone (Medium) or stimulated with LPS (0.1 ng/ml) alone (LPS) were included as controls. TNF-α in the culture supernatant was then quantitated using an ELISA kit and the results are expressed as mean concentration (pg/ml)±SD. These are representatives of 3 independent experiments.

In our previous study, rCRT/39-272 could effectively activate mouse macrophages in vitro [16]. Interestingly, rCRT/18-412 was as effective as rCRT/39-272 in inducing NO_2_^−^ production by mouse peritoneal macrophages in vitro, indicating that the presence, or deletion, of the C-domain did not affect the immunological activity of CRT (Fig. 2A). Purified nCRT was also able to activate macrophages in vitro, but with a 50-100-fold lower potency than rCRT/18-412 (Fig. 2A). Although rCRT/18-412 and rCRT/39-272 preparations had been repeatedly treated with polymyxin B (an efficient LPS inhibitor) prior to functional assays, the possibility that bacterial LPS might form a tight complex with rCRT, thereby resisting PMB treatment, was always a concern. Figs. 2B & 2C demonstrate that nCRT was not able to bind LPS in ELISAs, implying that formation of tight LPS-CRT complexes in the expressing *E. coli* cells is perhaps an unlikely event. Moreover, LPS and nCRT at sub-optimal concentrations did not show any synergistic effect in activating macrophages (Fig. 2D), providing circumstantial evidence against the “LPS contamination” hypothesis.

### Contaminating RPL2 does not Contribute to rCRT Activity

**Figure 3:**
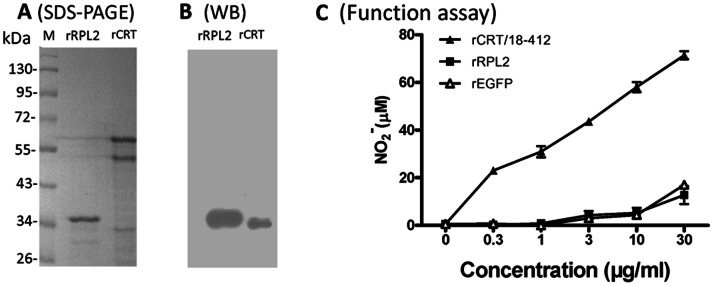
Identification and functional analysis of RPL2. rRPL2 and rCRT/18-412 were run in SDS-PAGE 12% gels followed by CBB staining (**A**) or WB using Cp32-Abs and HRP-labeled goat-anti-rabbit IgG (**B**). rCRT/18-412, rRPL2 and rEGFP were titrated against freshly isolated murine peritoneal macrophages for function analysis (**C**). After 24 h incubation, the concentration of NO_2_^−^ in the culture supernatant was determined by using Griess Reagent. The results are expressed as mean NO_2_^−^ concentration (µM)±SD.

**Figure 4:**
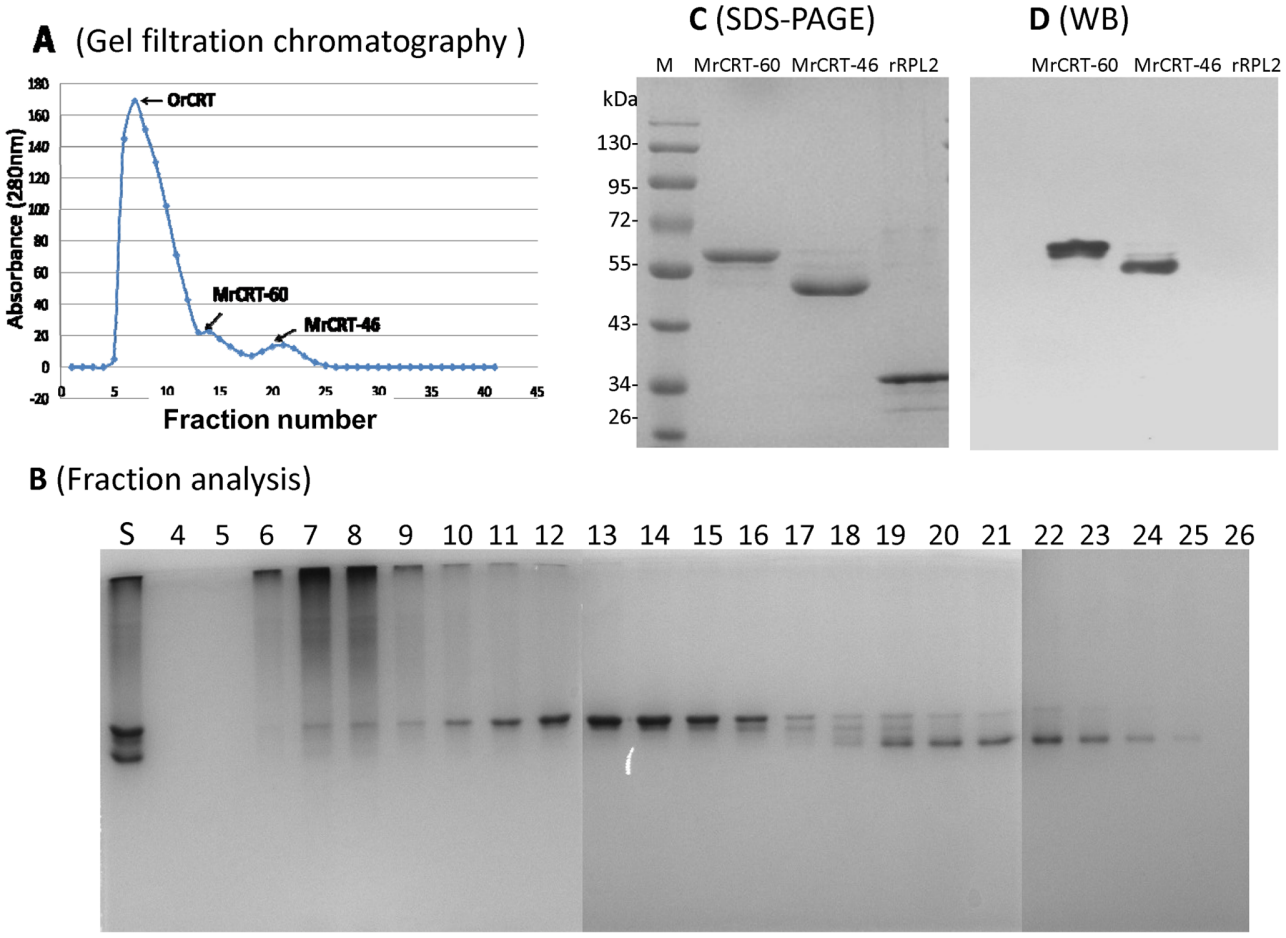
Separation of rCRT/18-412 monomers and oligomers by gel filtration. A sample of rCRT/18-412 (2 mls at 10 mg/ml concentration) was loaded on to a Sephadex G-75 column 80×2 cm, followed by elution using 0.9% NaCl at the speed of 20 ml/h collecting 2 ml fractions. Absorbance of the eluent at 280 nm wavelength was recorded (**A**) and all collected fractions were assayed using Native-PAGE (**B**). “S” refers to a sample of rCRT/18-412 (monomer and oligomer mixture) and “4–26” refers to fraction number. The resultant monomers MrCRT-60 kDa (Fractions 13–16) and MrCRT-46 kDa (Fractions 22–26) were further analyzed using CBB-stained SDS-PAGE (**C**) and WB employing polyclonal rabbit anti-CRT-Abs and HRP-labeled goat-anti-rabbit IgG (**D**). rRPL2 (right hand side lanes) was included as a specificity control. These are representatives of 3 independent experiments.

When preparing rCRT/18-412, Cp32 was a relatively persistent contaminant protein (Figs. 1 & 3). To characterize this protein, the Cp32 band was sliced out of SDS-PAGE gels and then (i) used to immunize C57/BL6 mice for preparation of specific antisera; and (ii) subjected to Q-TOF mass spectrometry analysis for sequence identification. The resultant antisera (Cp32-Abs) were able to recognize the immunizing protein band, but not the rCRT-60 kDa and rCRT-46 kDa bands, in WB (Fig. 3B). The MS result identified Cp32 as bacterial 50S ribosomal protein L2 (RPL2), which was confirmed by positive recognition of recombinant RPL2 (rRPL2, commercially available) by Cp32-Abs (Fig. 3B). It is noteworthy that rRPL2 was unable to activate macrophages, as evidenced in NO_2_^−^ production assays (Fig. 3C), and also that rRPL2 was not recognized by CRT-Abs (Figs. 4C & 4D). These results exclude the possibility that the contaminant Cp32 could make a substantial contribution to the immunological activities of rCRT.

### Isolation of rCRT Oligomers and Monomers

Since rCRT and nCRT differ in their ability to form oligomers in solution, we next asked if the potent immunological activities of rCRT polypeptides were the result of self-oligomerization. A Sephadex G-75 column was employed for fractionation of rCRT/18-412 oligomers and monomers. Fig. 4A shows that rCRT/18-412 was successfully separated into 3 peaks, designated sequentially as OrCRT (higher-molecular-weight rCRT/18-412 oligomers, major peak), MrCRT-60 kDa (monomeric rCRT/18-412, 60 kDa) and MrCRT-46 kDa (monomeric rCRT-46 kDa, aa18-386, see below). Guided by the SDS-PAGE results (Fig. 4B), we combined fractions 6–8 as OrCRTs, fractions 13–16 as MrCRT-60 kDa and fractions 19–26 as MrCRT-46 kDa. Subsequent Q-TOF MS analysis on samples of MrCRT-60 kDa and MrCRT-46 kDa, both of which were specifically recognized by CRT-Abs in WB (Figs. 4C & 4D), revealed their molecular mass as 46.78 kDa and 43.57 kDa, respectively. It can therefore be calculated that rCRT-46 kDa is a degradation product of rCRT-60 kDa less the C-terminal 26 amino acid residues.

### Immunological Activities of rCRT Oligomers and Monomers

**Figure 5:**
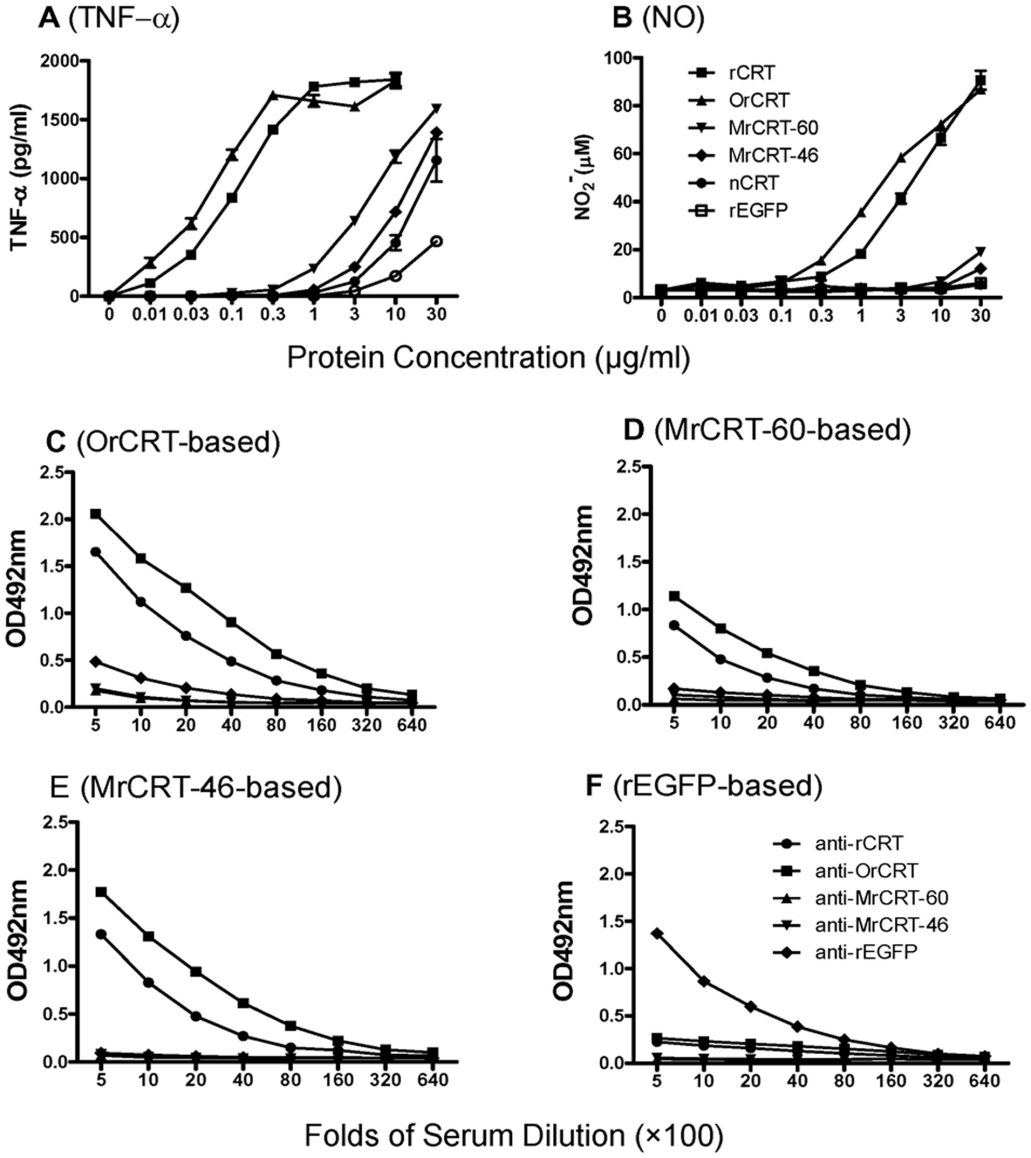
Functional comparison for rCRT monomers and oligomers. Samples of rCRT/18-412, OrCRT, MrCRT-60, MrCRT-46, nCRT and rEGFP were titrated against freshly prepared mouse peritoneal macrophages in 96-well plates. After 24 h incubation, concentrations of TNF-α(**A**) and NO_2_^−^ (**B**) in the culture supernatant were determined using ELISA kit or Griess reagent, respectively. For immunogenicity test, groups of C57/BL6 mice (5 per group) were s.c. injected with 100 µg of rCRT/18-412, OrCRT, MrCRT-60 kDa, MrCRT-46 kDa or rEGFP (in 100 µl PBS/mouse) and boosted with 50 µg of the antigen preparations a fortnight later. The mice were bled 10 days thereafter and their sera were assayed, in triplicate wells, in ELISAs based on OrCRT (**C**), MrCRT-60 kDa (**D**), MrCRT-46 kDa (**E**) or rEGFP (**F**). The detection Ab was HRP-conjugated goat-anti-mouse IgG with OPD as substrate, and the results are expressed as mean OD492 nm±SD. These are representatives of 3 independent experiments.

As shown in Fig. 5A, OrCRTs were modestly more effective than unfractionated rCRT/18-412 in eliciting TNF-α production by murine macrophages in vitro, while MrCRT-60, MrCRT-46 and nCRT were 50–100-fold less active than OrCRTs by comparison. A similar conclusion was also drawn when NO_2_^−^ production was taken as a measure for macrophage activation (Fig. 5B). Moreover, s.c. immunization of BALB/c mice with OrCRTs or unfractionated rCRT/18-412 (in the absence of adjuvant) elicited high titer serum IgG capable of recognizing both OrCRTs and MrCRTs in ELISAs (Figs. 5C–5F). By contrast, MrCRT-60 kDa (monomeric rCRT/18-412) and MrCRT-46 kDa (monomeric rCRT/18-386) were almost non-immunogenic in parallel experiments.

### Mapping the Immunological Activity Site (IAS) in CRT

**Figure 6:**
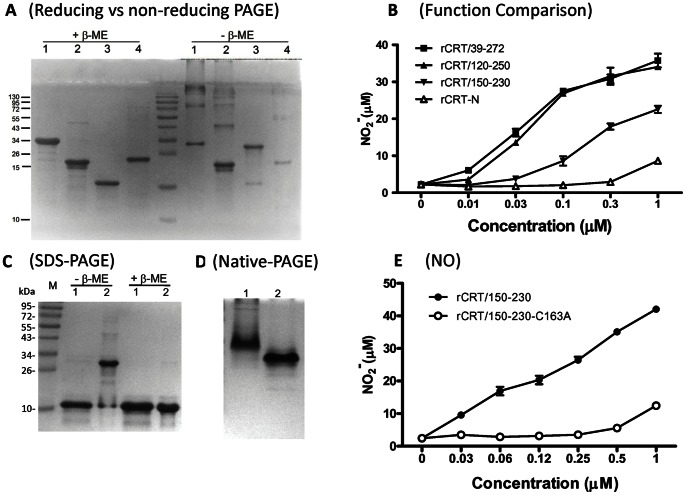
Mapping IAS in CRT. (**A**) rCRT fragments, including rCRT/39-272 (Lane 1), rCRT/120-250 (Lane 2), rCRT/150-230 (Lane 3) and rCRT-N (Lane 4), were analyzed using a SDS-PAGE 12% gel in the presence, or absence, of β-ME, and also titrated against peritoneal macrophages in 96-well plates (**B**). NO_2_^−^ in the culture supernatant was quantitated 24 h later by using Griess Reagent and the results are expressed as mean concentration (µM)±SD. rCRT/150-230 (Lane 1) and rCRT/150-230-C163A (Lane 2) were analyzed using a SDS-PAGE 12% gel in the presence, or absence, of β-ME (**C**) and using a native-PAGE (**D**). Freshly isolated murine peritoneal macrophages were stimulated with both fragments for 24 hrs. Concentrations of NO_2_^−^ (**E**) in the culture supernatant were determined using Griess reagent and the results are expressed as mean concentration (µM)±SD.

In order to assess whether the IAS is located in the N or P domain, the following fragments were designed: rCRT-N (residues 18–197, full length N domain), rCRT/120-250 (partial N, half P domains), rCRT/150-230 (partial N, one third P domains), rCRT/120-308 (partial N, full length P domain), and rCRT/198-308 (full length P domain). All, but rCRT/198-308 (very low yield and poor solubility), were successfully expressed in *E. coli* and affinity-purified. Both rCRT/120-250 and rCRT-N formed homodimers as well as higher-molecular-weight species (Fig. 6A). rCRT/120-250 was as potent as rCRT/39-272 and rCRT/18-412 in terms of eliciting NO_2_^−^ production by murine macrophages, while rCRT-N was almost completely inactive (Fig. 6B). Similar to rCRT/120-250, rCRT/120-308 also formed homodimers and higher-molecular-weight oligomers and showed strong macrophage-stimulatory activity in vitro (Table 1). rCRT/150-230, which contains a single cysteine residue (Cys163) and could only form homodimers (Fig. 6A), was approximately 10 fold less effective than rCRT/39-272 and rCRT/120-250 but nevertheless substantially more potent than MrCRTs and nCRT (Fig. 6B). A mutant form of rCRT/150-230 (namely rCRT/150-230-C163A) was prepared by substituting the cysteine residue at position 163 with alanine. The C163A mutant was neither able to form homodimers/oligomers in solution nor activate macrophages in vitro (Figs. 6C–6E). These results map the IAS of CRT to residues 150–230 and suggest that the N-domain probably does not contribute to the immunological activity of the molecule. Moreover, the importance of oligomerization for its immunological activities is further confirmed.

**Table 1:** Correlation between rCRT oligomerization and immunological activity.

CRT		Sequence	RMSAa)	Immunogenicityb)	Oligomerization statusc)
nCRT		Full length	1	–	Monomers
rCRT/18-412		aa18-412	50–100	+	Oligomers & monomers
OrCRT		aa18-412	50–100	+	Oligomers
MrCRT-60 kDa	aa18-412		3	–	Monomers, rCRT-60 kDa
MrCRT-46 kDa	aa18-386		1	–	Monomers, rCRT-46 kDa
rCRT/39-272	aa39-272		50–100	+	Mostly homodimers & oligomers
rCRT/120-250	aa120-250		50–100	+	Monomers, homodimers & oligomers
rCRT/120-308	aa120-308		50–100	+	Monomers, homodimers & oligomers
rCRT/150-230	aa150-230		10	ND	Monomers & homodimers
rCRT-N	aa18-197		<1	−/+	Monomers & oligomers

a) Relative macrophage stimulation activity (RMSA) of CRT is estimated using nCRT as reference, which is able to induce TNF-α production by macrophages in vitro at a concentration of 10 µg/ml or above (see Fig. 5A). The listed results are based on several batches of independent experiments including Fig. 5A.

b) Ability to elicit specific IgG responses in healthy BALB/c mice after s.c. immunization, in the absence of adjuvant, with 100 µg protein and a booster immunization with 50 µg protein a fortnight later. The mice were monitored for up to 28 days after the second immunization. ND: not detected; -: not immunogenic; +: strong humoral response; −/+: weak response.

c) As determined by Native-PAGE.

### OrCRTs Activate Macrophages in an Endocytosis-dependent Manner

**Figure 7:**
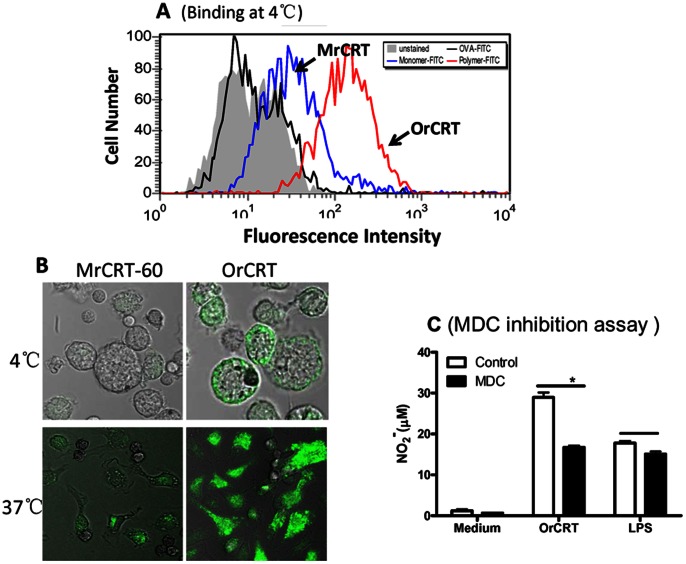
Specific binding and endocytosis of OrCRTs by macrophages. Freshly isolated mouse peritoneal macrophages were incubated in the presence, or absence, of FITC-OrCRT, FITC-MrCRT-60 kDa or FITC-OVA (15 µg/ml) for 30 min at 4°C, followed by flow cytometric analysis (**A**) and confocal laser scanning microscopy (**B**). The cells were also incubated at 37°C for 30 min and then observed under a confocal laser scanning microscope (**B**). For function assay, peritoneal macrophages were stimulated with either LPS or OrCRTs in the presence, or absence, of monodansylcadaverine (MDC) for 24 hrs (C). NO_2_^−^ in the supernatant was determined using Griess Reagent and the results expressed as mean concentration±SD. These are representatives of 3 independent experiments. *: *p*<0.05 comparing with sample without MDC.

In order to investigate the mechanisms for the relationship between rCRT oligomerization and its potent immunological activities, fractionated OrCRTs and MrCRT-60 kDa (monomeric rCRT/18-412) were conjugated with FITC and then compared for ability to stain macrophages. After 30 min incubation at 4°C, FITC-OrCRTs showed stronger binding to murine macrophages than FITC-MrCRTs, as evidenced by flow cytometric analysis and confocal laser scanning microscopy (Fig. 7A&B). Substantially more FITC-OrCRTs than FITC- MrCRTs were endocytosed by macrophages after 30 min incubation at 37°C (Fig. 7B). Moreover, monodansylcadaverine (MDC), an endocytosis inhibitor [21], partially suppressed NO_2_^−^ production by macrophages under stimulation with OrCRTs, while LPS-triggered macrophage activation was unaffected by the same treatment (Fig. 7C).

## Discussion

In this study, we have examined different hypotheses (i.e. C-domain deletion hypothesis, LPS contamination hypothesis, RPL2 hypothesis and oligomerization hypothesis) for explanation of the much stronger immunogenicity and immunostimulatory activities of rCRT than nCRT. Our data strongly suggests that self-oligomerization of the rCRT polypeptides is a key factor for their strong immunological activities. As summarized in Table 1, all four rCRT fragments containing residues 120–250 (including rCRT/18-412, rCRT/39-272, rCRT/120-250 and rCRT/120-308) self-oligomerized and exhibited potent macrophage activating ability in vitro and strong immunogenicity in vivo, while nCRT, which existed mainly in monomeric form, showed only modest stimulatory activities towards macrophages and was non-immunogenic in mice (Figs. 1 & 3). Moreover, fractionated rCRT/18-412 oligomers were 50–100 folds more active than MrCRTs in activating macrophages in vitro (Fig. 5). The N domain polypeptide (rCRT/18-197) also formed higher-molecular-weight oligomers (Fig. 6A), but it is almost completely inactive in terms of stimulating macrophages in vitro (Fig. 6B) and inducing Ab responses in vivo (Table 1). Clearly, oligomerization is necessary but not sufficient to arm the rCRT polypeptides with potent immunological activities.

In general, oligomerized (aggregated) proteins are more immunogenic than their monomeric counterparts. However, the immunogenicity of OrCRTs is by far the most impressive and not comparable by other protein aggregates. Even in the absence of any adjuvant, minute amount (1 ng/mouse) rCRT/18-412 or rCRT/39-272 can elicit strong IgG responses in mice (Fig. 2; Ref. 16). Most, if not all, other protein antigens are unable to induce IgG production in T-cell-deficient nude mice, yet rCRT/39-272 and rCRT/18-412 could do so relatively efficiently [16], [22]. Moreover, the potent adjuvanticity of the rCRT polypeptides is also quite phenomenal. For instance, rCRT/39-272 (mostly in oligomeric forms) is able to assist the production of IgG Abs against fused target proteins or conjugated polysaccharides in healthy mice or T-cell-deficient nude mice [17], [22].

Dimerization/oligomerization of soluble CRT was also observed by previous investigators. For example, Jorgensen et al documented that shielding of the free Cys163 in the N domain is the main reason that nCRT exists mainly in monomeric form under physiological conditions. Under partial unfolding conditions such as high temperature or low PH, however, the free Cys could be exposed and subsequently help CRT oligomerization [23]. nCRT (isolated from human placenta) formed homodimers and higher-molecular-weight species through disulfide bonding as well as non-covalent association, and that oligomerized nCRT showed higher binding affinity to peptides and denatured proteins [23]. In the case of prokaryotically expressed rCRT polypeptides (the folding of which may differ from nCRT), all Cys residues in sequence could contribute to its olimerization, although it might not be absolutely necessary that all 3 Cys residues have to be available for inter-molecular cross-linking at the same time. Mancino and colleagues illustrated that self-oligomerized rCRT could better assist HLA folding in vitro [24]. There are 3 conserved cysteine residues in the amino acid sequence of CRT. Cys105 and Cys137 form intramolecular disulfide bonds, while Cys163 is free [25]-[26]. It is likely that rCRT polypeptides are unable to form appropriate intra-molecular disulfide bonds like in nCRT, thereby allowing all 3 cysteine residues to participate in self-oligomerization, although formation of higher-molecular-weight oligomers could also occur through non-covalent association of CRT [23], [27]. CRT is considered one of the heat shock proteins (HSPs) that share many immunological and biochemical activities [28]-[29]. Interestingly, self-oligomerization also occurs to other HSPs such as GRP94 and HSP90, which is likely associated with their chaperone function [30]–[32].

Koslov et al and Chouquet et al have recently solved the crystal structure of the lectin site as well as a peptide-binding site in the CRT N domain [18], [19], which apparently play important roles in the physiological function of CRT. However, our data maps the IAS of CRT to a region of 80 residues between aa150–230 (Fig. 6). As rCRT-N was almost completely inactive in functional assays (Fig. 6, Table 1), the IAS may be narrowed down further to aa198–230 in the P domain, although a series of truncated synthetic peptides covering this region would be needed for a concrete conclusion. Interestingly, this sequence of 30 amino acid residues contains the RA shared epitope (SE)-binding site (residues 217–224) of CRT recently mapped by Ling et al. [33]. Such coinciding results from 2 independent groups further emphasize the importance of the aa198–230 region of the P domain to the immunological activities of CRT. It has been documented that the CRT P domain adopts a hairpin-shaped structure, its sequence is consisted of 3 copies of a repeat motif (type 1: IXDPXXXKPEDWD) followed by 3 copies of another repeat motif (type 2) [34]–[36]. Interestingly, the SE-binding site almost completely overlaps the type 1 motif [33]. It is reasonable to suggest that the type 1 motif might also represent the core of IAS of CRT, responsible for direct biding with activation receptors on the surface of immune cells. Functional comparison data showed that rCRT/120-308 and rCRT/120-250 (possessing 3 type 1 repeats, the latter without type 2 repeats) are 10 times more active than rCRT/120-230 (with 2 type 1 repeats, no type 2 repeats) in activating macrophages (Table 1, Fig. 6B), implying that (i) type 2 repeats do not contribute to the immunological activity of the molecule; and (ii) the presence of 3 copies of the type 1 motif is of crucial importance for the potent immunological function of OrCRTs. It can be envisaged that oligomerization of CRT multiplies its binding avidity to immune cells with receptors for the type 1 motif, thereby enabling it to deliver stimulatory signals to the cells in a highly efficient manner.

We further predict that the high-avidity binding of OrCRTs to macrophages may easily trigger their uptake process. Indeed, OrCRTs can activate macrophages in an endocytosis-dependent pathway (Fig. 7). Perhaps OrCRTs could use certain intracellular sensors to deliver immunostimulatory activity to the responding immune cells. An example of an intracellular sensor for endocytosed polymeric proteins is the NOD-like receptor (NLR) protein NLRP3, a key component of the NLRP3 inflammasome and an important intracellular sensor for microbial ligands and endogenous danger signals [37]. Masters and colleagues demonstrated that soluble oligomers of islet amyloid polypeptide (IAPP), a protein that forms amyloid deposits in the pancreas during type 2 diabetes, could be endocytosed and trigger the NLRP3 inflammasome and generate mature IL-1β [38].

Finally, the region of aa150-230 of CRT has a 98% homology between mouse and human. It would be of interest to examine if there is a functional relationship between the SE binding site and the IAS of CRT. Irrespective of such a relationship, oligomerization might occur to extracellular CRT released by tissue cells thereby converting CRT into a highly active form, which may play important roles in the development and pathogenesis of autoimmune disorders in humans.

## Materials and Methods

### Purification of nCRT

nCRT was purified from mouse livers using a modification of a previously described methods [39], [40]. Briefly, fresh mouse liver cells (erythrocytes depleted) were collected and centrifuged at 1200 rpm for 5 min. The cell pellet was lysed in 3 volumes of lysis buffer (1% Triton-X 100, 0.2 mM PMSF in PBS) for 30 min on ice, followed by centrifugation at 35,000 g for 60 minutes. The supernatant was then precipitated using (NH_4_)_2_SO_4_ and the final precipitate dissolved in binding buffer (150 mM NaCl, 20 mM Tris, PH7.4) followed by dialysis against this buffer. The sample was applied to a DEAE Sephadex A50 column (10×2 cm, *GE* Healthcare, US) which was then sequentially washed with binding buffer and washing buffer (280 mM NaCl, 20 mM Tris, PH7.4) at 1 ml/min to remove contaminating proteins. The fractions were eluted with a linear salt (280–500 mM NaCl) gradient.

### Recombinant Proteins

The preparation of rCRT/39-272 and rEGFP was as previously described [16] and the other rCRT polypeptides used in this study were prepared using the same prokaryotic system. Specific primers were as follows: rCRT/18-412, sense 5′-AAGCTTTTG GCCAGGGGATTCTTCCTCA-3′, anti-sense 5′-AAGC TTTTGGCCAGGGGATTCTTCCTCA-3′; rCRT/39-272, sense 5′-CGCGGATCCGAATCCAAACATAAGTCCGATTTTGG-3′, anti-sense 5′-CCCAAGCTTTTTTCAGGATTTTGAATCACTGGTG-3′; rCRT–N, sense 5′-CGGATCCGACCCTGCCATCTATTTCAAAGA-3′, anti-sense 5′-CCCAAGCTTCTACTCCAAGGAGCCTGACTC-3′; rCRT/120-250, sense 5′-CGGATCCAAGGACATGCATGGAGACTCAGA-3′, anti-sense 5′-CCCAAGCTTCTAAGGCTTCTTAGCATCAGGGT-3′; rCRT/150-230, sense 5′-CGGATCCTACAAGGGCAAGAATGTGCTGAT-3′, anti-sense 5′-CCCAAGCTTCTAATCTGTGGGGTCATCGATCTTG-3′. The rCRT/150-230-C163A mutant was constructed using TaKaRa Mutan BEST Kit (TaKaRa Biotechnology Co. Ltd) following manufacturer’s instruction. The mutant primer was as follows: sense: 5′-ATTCACACACCTATACACACTGATT-3′, antisense: 5′-TCATCATCCTTAGCCCGGATATCCT-3′. All proteins were desalted by passing through PD10 columns (Pierce, Rockford, IL, USA). Protein concentration was determined using Coomassie protein assay reagent (Pierce, Rockford, IL, USA). All recombinant proteins were used at over 90% purity as judged by CBB-stained SDS-PAGE gels.

### Western Blotting

The separated protein bands in SDS-PAGE gels were electro-blotted onto PVDF membranes, at a constant current of 250 mA in transbuffer (50 mM Tris, pH 8.0, containing 0.192 M glycine and 20% methanol), using a Bio-Rad Trans-Blot Cell. The strips were incubated for 1 hr at room temperature in blocking buffer (TBS containing 5% nonfat milk), followed by a overnight incubation at 4°C with constant agitation in indicated antibody diluted in blocking buffer. After 3 washes with TBS containing 0.05% Tween 20, strips were incubated for 1 hr with HRP-conjugated secondary antibody (Southern Biotechnology Associates Inc., USA) and visualized using the ECL detection system as recommended by the manufacturer (Applygen Technologies Inc., Beijing, China).

### ELISAs

LPS-based and CRT-based ELISAs were as previously described [41]. Briefly, ELISA plates were coated at 4°C overnight with rCRT or LPS and subsequently incubated with blocking solution (1% BSA in PBS) for 2 hrs at 37°C. The wells were washed five times with PBS containing 0.05% Tween 20 (PBS-T) prior to incubation at 37°C with 100 µl of diluted mouse sera or with indicated protein (nCRT and lactoferrin) followed by corresponding antibody in triplicate. After 5 washes with PBS-T, the plates were further incubated with HRP-labeled goat-anti-mouse or goat-anti-rabbit IgG Abs (Southern Biotechnology Associates Inc., AL., USA) for 1 hr at 37°C. The reaction was developed with 100 µl of *o*-phenylenediamine (OPD, Sigma) for 5 min and stopped with 100 µl 2 M H_2_SO_4_. Optical density (OD) was measured at 492 nm in an ELISA spectrophotometer (Titertek Multiscan Plus MK II; ICN Flow Laboratories, Irvine, UK).

### Cells and Tissue Culture

All cells were cultured in complete R10 medium: RPMI-1640 supplemented with 10% (v/v) fetal bovine serum (Hyclone, USA), penicillin/streptomycin (100 U/ml), L-glutamine (2 mM), and β-ME (5×10^−5^ M). For preparation of mouse peritoneal macrophages, mice were injected i.p. with 3% thioglycollate (1 ml/mouse) and the macrophages retrieved from the peritoneum 3 days later using a syringe. The resultant cells were>90% positive for F4/80 marker, as determined by FACS analysis.

### Macrophage Activation Assays

Freshly prepared C57/BL6 mouse peritoneal macrophages (1.5×10^5^ cells/well) were stimulated with rCRT fragments, or nCRT, or LPS, in R10 medium in 96-well tissue culture plates for 24 hrs in a 5% CO_2_ incubator at 37°C. The concentration of TNF-α in the culture supernatant was determined using ELISA kits (Biolegend, San Diego, USA) following the manufacturer’s instructions. The concentration of NO_2_^−^ in the supernatant was determined by Griess Reagent. Standard curves were established using NaNO_2._

### Mice and their Immunization

Female C57BL/6 mice between the age of 6–8 weeks were purchased from the Model Animal Research Center, Nanjing, China. All animals were maintained under specified-pathogen-free (SPF) conditions and animal usage was conducted according to protocols approved by the Soochow University Institutional Animal Care and Use Committee. For immunization with rCRT, mice were immunized s.c. at the base of the tail with 100 µg protein in total 100 µl PBS. When booster immunization was needed, 50 µg of protein in 200 µl PBS was injected intraperitoneally (i.p.). For immunization with PAGE gel slices containing Cp32, the band was cut out with a razorblade and then frozen in liquid nitrogen and emulsioned with CFA, which was then injected s.c. into female C57/BL6 mice. Serum samples were collected by tail bleeding, aliquoted and kept at −20°C until use.

### Separation of OrCRTs and MrCRTs

A Sephadex G-75 (GE Healthcare, US) column of 80×2 cm was employed. 5 ml of rCRT/18-412 at 10 mg/ml was loaded into the column, followed by elution with 0.9% NaCl at 20 ml/h and collected every 2 ml.

### FITC Labeling of Proteins

Protein was labeled by FITC using FluoroTag™ FITC Conjugation Kit (Sigma, US) according to the manufacturer’s instructions. In brief, 1 mg of protein was dialyzed against 0.1 M Na_2_CO_3_, pH 9.5 at 5 mg/ml. Fluoresceine isothiocyanate (FITC, Sigma) was dissolved in the same buffer with DMSO at 1 mg/ml and 50 µl of this solution was added to the protein. The sample was gently mixed for 2 h at RT. Separation of labeled protein from unbound FITC was performed on a Sephadex G-25 column.

### Flow Cytometric Analysis

10^6^ freshly isolated peritoneal macrophages were stained with APC-anti-F4/80 and then incubated with 15 µg/ml of FITC-OrCRT, FITC-MrCRT or FITC-OVA for 30 min at 4°C. The binding of CRT by macrophages was determined by flow cytometry (BD FACS Canto II, US).

### Confocal Laser Scanning Microscopy

Macrophages (1.5×10^5^/well) were plated onto poly-l-lysine coated glass slides and allowed to adhere. The cells were then incubated in a total volume of 200 µl 0.5% BSA in PBS with 15 µg/ml FITC-OrCRT, FITC-MrCRT or FITC-OVA for 30 min at 4°C or 37°C. After washing to remove unbound protein, macrophages were fixed in 1% paraformaldehyde and stored at 4°C until microscopic analysis. Finally, 1% of glycerol was added and slides were counterstained with 1 mg/ml DAPI. The cells were imaged with a Nikon confocal microscope system A1.

### Statistical Analysis

All experiments were repeated at least 3 times and the results are expressed as mean±standard deviation of the mean (SD). Statistical analysis was performed using the Independent-Samples *t* test or two-side paired *t* test between groups using the SPSS 14.0 program (SPSS, Chicago, IL). Differences were considered statistically significant at *p*<0.05.
